# Supplementary material for: Sorghum Metabolic Atlas: A fluorescent protein‐based subcellular localization resource for sorghum enzymes
Source: Plant J. 2026 Oct 29;128(3):e71166. doi: 10.1111/tpj.71166 (PMC13720221; doi:10.1111/tpj.71166)
Supplement: Supplementary file 1 [file TPJ-128-0-s002.pdf]

Supporting information

Establishment of a protoplast transformation system in *Sorghum bicolor*

To enable large-scale fluorescent protein (FP)-based subcellular localization in *Sorghum bicolor* (sorghum), we established a method for robust protoplast isolation and transient transformation. Although FP-based transformation of protoplasts has been performed in various plant species (Gao et al., 2019; Lin et al., 2018; Trinidad et al., 2021; Wu & Hanzawa, 2018; Yang et al., 2022; Zhang et al., 2011), its implementation in sorghum remains limited (Meng et al., 2020). We optimized a protocol adapted from (Birnbaum et al., 2005; Gomez-Cano et al., 2019; Shulse et al., 2019). Protoplast viability was assessed using fluorescein diacetate (FDA) (Figure S3a), which detects esterase activity converting non-fluorescent FDA into fluorescent fluorescein in intact cells (Jones et al., 2016). Transformation efficiency was evaluated using a mitochondrion marker (TIM21 (1–50)-Citrine) driven by the CaMV35S promoter (Osaki & Kodama, 2017) (Figure S3b). Transformation efficiency ranged from 17% to 36% across three biological replicates (Figure S3c), yielding abundant protoplasts for optical screening.

To assess subcellular localization of sorghum enzymes to organelles, we assembled a panel of FP-tagged markers for major organelles, including the nucleus, cytosol, Golgi, endoplasmic reticulum (ER), mitochondria, peroxisomes, and lipid droplets. When expressed in sorghum protoplasts, subcellular localization of these markers closely resembled those observed in the plant species from which they were derived (Figure S3d). In the case of Golgi apparatus, co-localization of two enzymes with Arabidopsis orthologs that are localized to Golgi led us to select one of the enzymes as a Golgi marker (Figure S3d). Chloroplasts were readily identified by overlapping chloroplast markers and chlorophyll autofluorescence (Figure S3e). The optimized protoplast platform provides a consistent and effective approach for FP-based subcellular localization analysis in sorghum.

References

Birnbaum, K., Jung, J.W., Wang, J.Y., Lambert, G.M., Hirst, J.A., Galbraith, D.W. et al. (2005) Cell type-specific expression profiling in plants via cell sorting of protoplasts from fluorescent reporter lines. *Nature Methods*, 2(8), 615–619.

Gao, L., Shen, G., Zhang, L., Qi, J., Zhang, C., Ma, C. et al. (2019) An efficient system composed of maize protoplast transfection and HPLC-MS for studying the biosynthesis and regulation of maize benzoxazinoids. *Plant Methods*, 15(1), 144.

Gomez-Cano, L., Yang, F. and Grotewold, E. (2019) Isolation and efficient maize protoplast transformation. *Bio-protocol*, 9(16), e3346.

Jones, K., Kim, D.W., Park, J.S. and Khang, C.H. (2016) Live-cell fluorescence imaging to investigate the dynamics of plant cell death during infection by the rice blast fungus Magnaporthe oryzae. *BMC Plant Biology*, 16(1), 69.

Lin, C.-S., Hsu, C.-T., Yang, L.-H., Lee, L.-Y., Fu, J.-Y., Cheng, Q.-W. et al. (2018) Application of protoplast technology to CRISPR/Cas9 mutagenesis: from single-cell mutation detection to mutant plant regeneration. *Plant Biotechnology Journal*, 16(7), 1295–1310.

Meng, R., Wang, C., Wang, L., Liu, Y., Zhan, Q., Zheng, J. et al. (2020) An efficient sorghum protoplast assay for transient gene expression and gene editing by CRISPR/Cas9. *PeerJ*, 8, e10077.

Osaki, Y. and Kodama, Y. (2017) Particle bombardment and subcellular protein localization analysis in the aquatic plant *Egeria densa*. *PeerJ*, 5, e3779.

Shulse, C.N., Cole, B.J., Ciobanu, D., Lin, J., Yoshinaga, Y., Gouran, M. et al. (2019) High-throughput single-cell transcriptome profiling of plant cell types. *Cell Reports*, 27(7), 2241–2247.e4.

Trinidad, J.L., Longkumer, T. and Kohli, A. (2021) Rice protoplast isolation and transfection for transient gene expression analysis. *Methods in Molecular Biology,* 2238, 313–324.

Wu, F. and Hanzawa, Y. (2018) A simple method for isolation of soybean protoplasts and application to transient gene expression analyses. *Journal of Visualized Experiments, (*131), e57258.

Yang, D., Zhao, Y., Liu, Y., Han, F. and Li, Z. (2022) A high-efficiency PEG-Ca2+-mediated transient transformation system for broccoli protoplasts. *Frontiers in Plant Science*, 13, 1081321.

Zhang, Y., Su, J., Duan, S., Ao, Y., Dai, J., Liu, J. et al. (2011) A highly efficient rice green tissue protoplast system for transient gene expression and studying light/chloroplast-related processes, *Plant Methods*, 7(1), 30.
