# Supplementary material for: Sorghum Metabolic Atlas: A fluorescent protein‐based subcellular localization resource for sorghum enzymes
Source: Plant J. 2026 Oct 29;128(3):e71166. doi: 10.1111/tpj.71166 (PMC13720221; doi:10.1111/tpj.71166)
Supplement: Supplementary file 2 [file TPJ-128-0-s004.pdf]

Supporting Figures

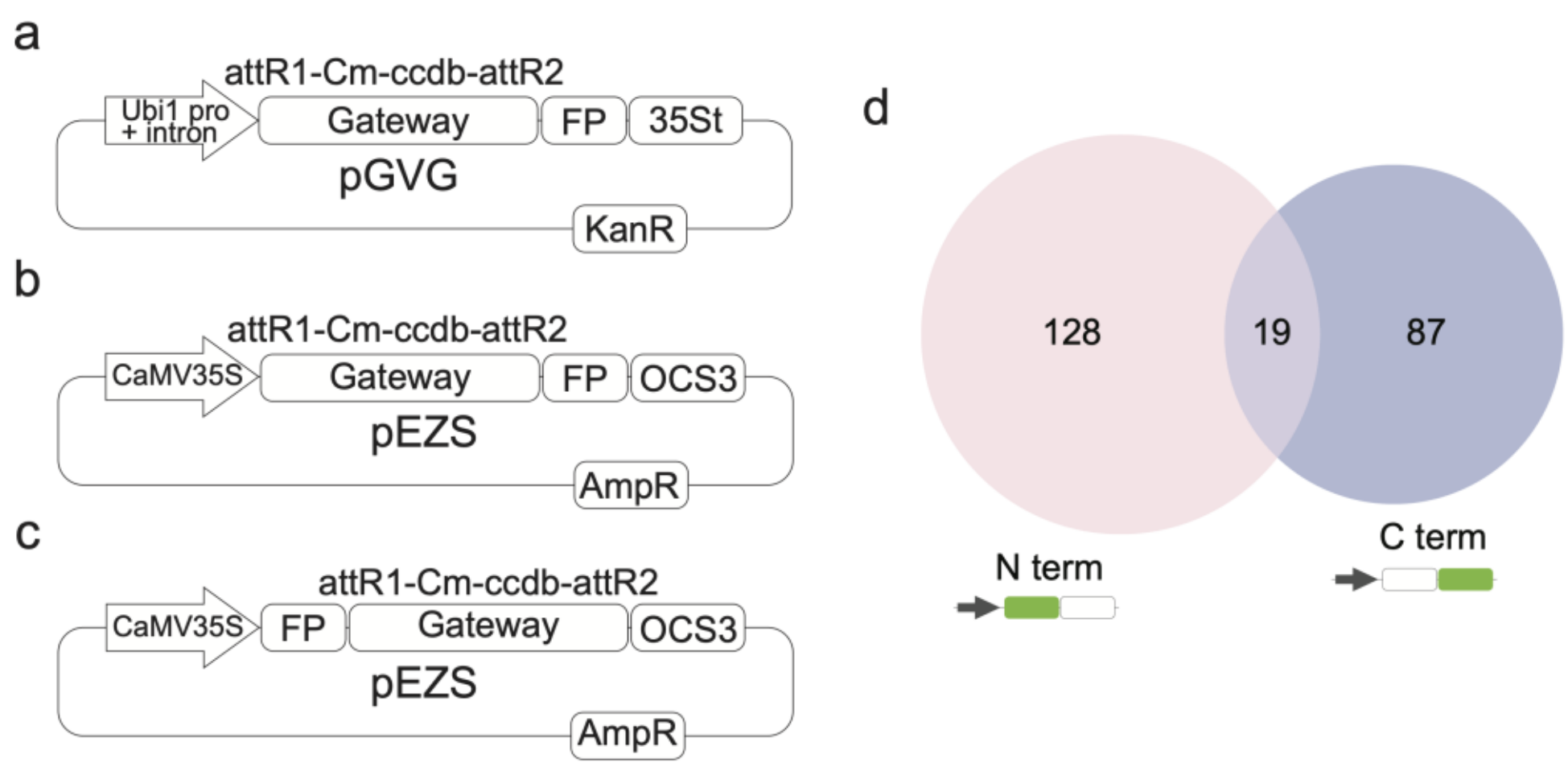

Figure S1: Vector design and comparison of N and C terminal fusion constructs.

a-c) Schematic diagrams of Gateway-compatible binary vectors used for subcellular localization in sorghum protoplasts. a) pGVG vector containing the maize Ubiquitin-1 promoter with intron, C-terminal mEGFP tag, and 35S terminator, conferring kanamycin resistance. b,c) pEZS vector containing CaMV 35S promoter, FP fusion at (b) C-terminal or (c) N-terminal, OCS3 terminator, conferring ampicillin resistance.

d) Venn diagram showing sorghum enzymes tagged with fluorescent proteins at the N- or C-terminus for localization analysis.

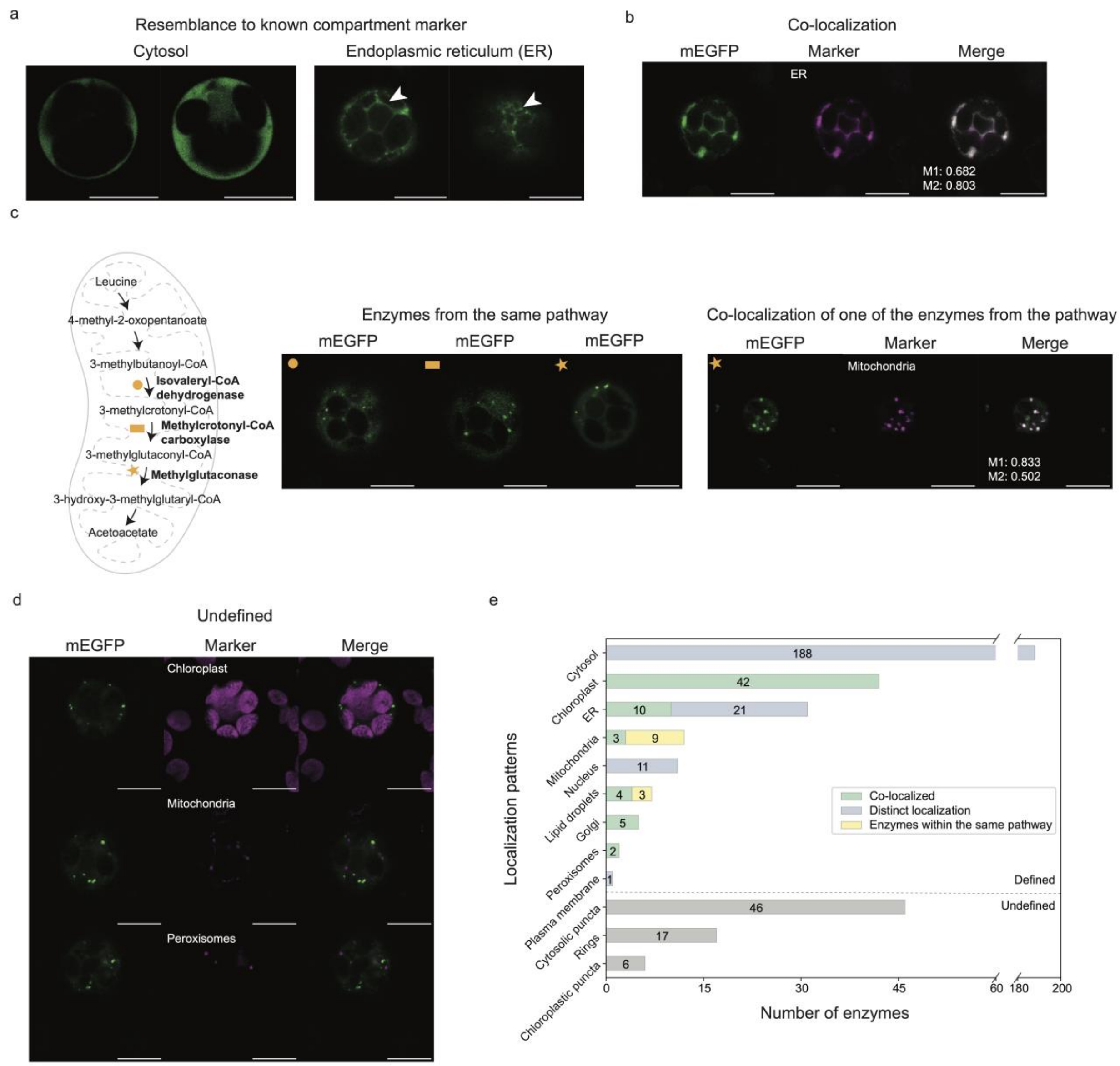

Figure S2: Assigning subcellular compartments based on localization patterns.

a) Representative images of patterns of fluorescent-tagged enzymes showing cytosolic (Sobic.008G179900) or endoplasmic reticulum (Sobic.004G064000). 2 sections from the z-stack are shown for each enzyme.

b) Representative image of co-localization of GFP-tagged enzyme (Sobic.001G195100) with an ER-marker, confirming ER localization.

c) Schematic of leucine degradation pathway with analyzed enzymes highlighted (in bold) include Sobic.009G027000, Sobic.007G130600, and Sobic.004G297400.

d-e) Highlighted enzymes show puncta and weaker cytosolic signals. Methylcrotonyl-CoA carboxylase co-localizes with the mitochondrial marker, confirming mitochondrial localization.

f) Enzymes with undefined cytosolic puncta and rings are shown via localization patterns of

Cysteine Desulfurase (CD-Sobic.004G132600) and Succinate Semialdehyde Dehydrogenase (SSADH-Sobic.004G058600), respectively.

g) Representative images of CD (green) with mitochondrial (top-middle) or peroxisomal (bottom-middle) markers. The lack of overlap indicates that CD cytosolic puncta does not correspond to the tested marker and may represent an uncharacterized compartment. Scale bars represent 10 μm.

h) Distribution of enzyme localization based on supporting evidence, including distinct localization, co-localization, and pathway association.

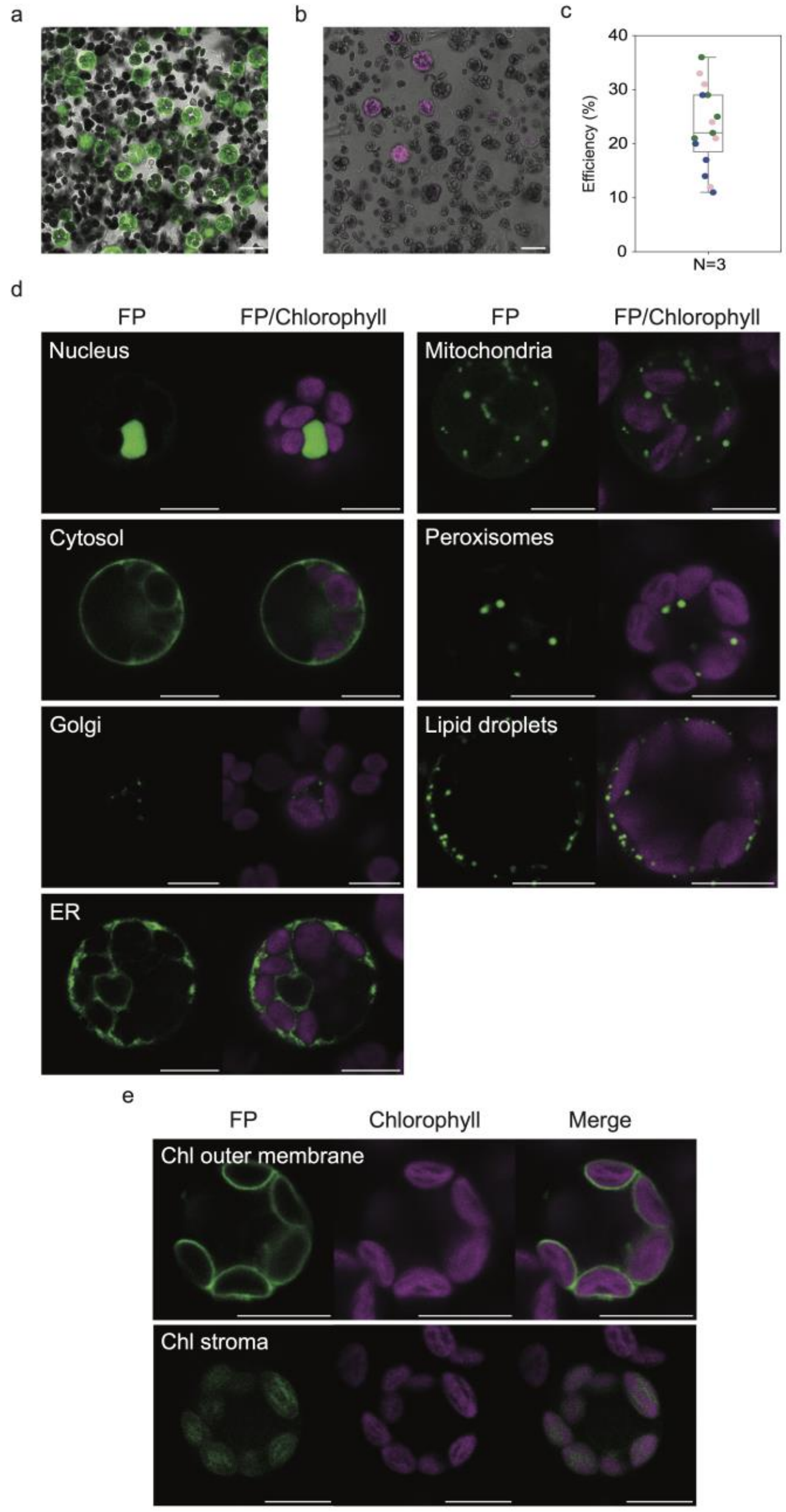

Figure S3: Validation of subcellular localization markers in sorghum leaf protoplasts.

a) Fluorescein diacetate (FDA) staining of untransformed protoplasts. Viable protoplasts appeared as spherical, green-fluorescing cells, indicating membrane integrity.

b) Representative confocal images of protoplasts transiently transformed with a mitochondrial marker (pGWTCaMV35S-Citrine-TIM21(1-50) from Osaki et al., 2017. Transformed cells expressing mitochondrial-localized Citrine are indicated by magenta merged on a bright field image.

c) Quantification of transformation efficiency from five randomly captured images showed a range of 17-36% across three independent experiments. Each dot represents one image.

d) FP-tagged markers for various organelles, including nucleus (pGWTCaMV35S-Citrine-NLS), cytosol (pGWTCaMV35S-Citrine), Golgi (pEZS-Sobic.004G326900-GFP as marker), endoplasmic reticulum (ER) (pEZS-αAmySP-mCherry-HDEL), mitochondria (pGWTCaMV35S-TIM21(1-50)-Citrine), peroxisomes (pEZS-mCherry-PTS), and lipid droplets (Nile Red stain). Merged images display FP signals alongside chlorophyll autofluorescence where relevant.

e) Fluorescent protein (FP) localization in the chloroplast outer membrane (pGWTCaMV35S-OEP7-mCherry) and stroma (pGWTCaMV35S-RBCS1a-RFP). FP signals (green) are shown separately and merged with chlorophyll autofluorescence (magenta).

Scale bars represent 20 μm in panels a) and b), 10 μm in panels d) and e). Fluorescent protein signal colors have been adjusted to green for visualization.
